# Global regulation of gene expression in response to cysteine availability in *Clostridium perfringens*

**DOI:** 10.1186/1471-2180-10-234

**Published:** 2010-09-07

**Authors:** Gaelle André, Elise Haudecoeur, Marc Monot, Kaori Ohtani, Tohru Shimizu, Bruno Dupuy, Isabelle Martin-Verstraete

**Affiliations:** 1 Institut Pasteur, Unité de Génétique des Génomes Bactériens and Unité des Bactéries Anaérobies et Toxines, 28 rue du Docteur Roux, 75015 Paris, France; 2 CNRS URA 2171, 75015 Paris, France; 3 Institut Pasteur, Laboratoire de Pathogénèse des Bactéries Anaérobies, 28 rue du Docteur Roux, 75015 Paris, France; 4 Université Paris 7-Denis Diderot, 75205 Paris, France; 5 Department of Bacteriology, Graduate School of Medical Science, Kanazawa University, Ishikawa, 920-8640 Japan

## Abstract

**Background:**

Cysteine has a crucial role in cellular physiology and its synthesis is tightly controlled due to its reactivity. However, little is known about the sulfur metabolism and its regulation in clostridia compared with other firmicutes. In *Clostridium perfringens*, the two-component system, VirR/VirS, controls the expression of the *ubiG *operon involved in methionine to cysteine conversion in addition to the expression of several toxin genes. The existence of links between the *C. perfringens *virulence regulon and sulfur metabolism prompted us to analyze this metabolism in more detail.

**Results:**

We first performed a tentative reconstruction of sulfur metabolism in *C. perfringens *and correlated these data with the growth of strain 13 in the presence of various sulfur sources. Surprisingly, *C. perfringens *can convert cysteine to methionine by an atypical still uncharacterized pathway. We further compared the expression profiles of strain 13 after growth in the presence of cystine or homocysteine that corresponds to conditions of cysteine depletion. Among the 177 genes differentially expressed, we found genes involved in sulfur metabolism and controlled by premature termination of transcription via a cysteine specific T-box system (*cysK*-*cysE*, *cysP1 *and *cysP2*) or an S-box riboswitch (*metK *and *metT*). We also showed that the *ubiG *operon was submitted to a triple regulation by cysteine availability via a T-box system, by the VirR/VirS system via the VR-RNA and by the VirX regulatory RNA.

In addition, we found that expression of *pfoA *(theta-toxin), *nagL *(one of the five genes encoding hyaluronidases) and genes involved in the maintenance of cell redox status was differentially expressed in response to cysteine availability. Finally, we showed that the expression of genes involved in [Fe-S] clusters biogenesis and of the *ldh *gene encoding the lactate dehydrogenase was induced during cysteine limitation.

**Conclusion:**

Several key functions for the cellular physiology of this anaerobic bacterium were controlled in response to cysteine availability. While most of the genes involved in sulfur metabolism are regulated by premature termination of transcription, other still uncharacterized mechanisms of regulation participated in the induction of gene expression during cysteine starvation.

## Background

Sulfur is a crucial element for cysteine and methionine, and is also present in several coenzymes and cofactors (thiamine, biotin, lipoic acid, coenzyme A and coenzyme M). Cysteine is important in the biogenesis of iron-sulfur ([Fe-S]) clusters [1], is found in the catalytic site of several enzymes and also aids protein folding and assembly by forming disulfide bonds. Cysteine-containing molecules such as thioredoxin, glutaredoxin, glutathione, mycothiol or bacilithiol are also important in protecting cells against oxidative stress [2-4]. Methionine, the universal initiator of protein synthesis, is also a key factor in various cellular functions. Its derivatives, S-adenosylmethionine (SAM) and autoinducer 2 (AI-2), are involved in several cellular processes including methylations and polyamine biosynthesis for SAM and quorum sensing and gene regulation for AI-2 [5].

**Figure 1:**
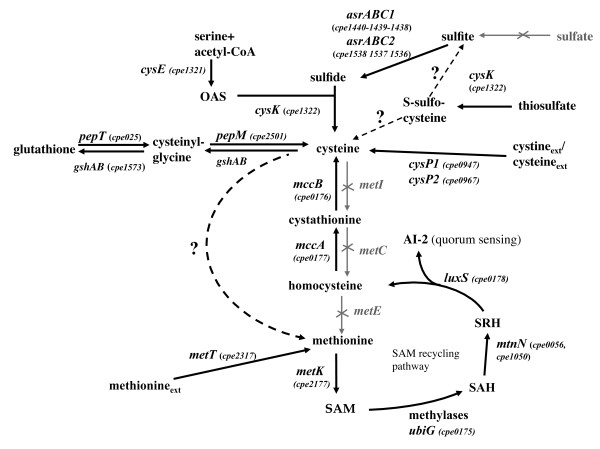
**Reconstruction of sulfur metabolism in *C. perfringens***. We used the genomic data, growth assays and expression profiling to propose a tentative reconstruction of sulfur metabolism in *C. perfringens*. The *cpe *numbers for *C. perfringens *genes (strain 13) correspond to those of ClostriDB http://xbase.bham.ac.uk/clostridb/. The genes were renamed according to *B. subtilis *orthologues. The steps present in *B. subtilis *but absent in *C. perfringens *(sulfate assimilation and methionine biosynthesis by transsulfuration) are indicated by grey crossed arrows. A dotted arrow indicated the possible existence of a pathway. "?" indicates a step or a pathway for which a gene is lacking or remains to be identified. Serine *O*-acetyltransferase, *cysE*; OAS-thiol-lyase, *cysK*; anaerobic sulfite reductase, *asrABC*; glutamate-cysteine ligase/glutathione synthetase, *gshAB *; SAM synthase, *metK*; adenosyl-homocysteine nucleosidase, *mtnN*; S-ribosyl-homocysteine lyase, *luxS*; cystathionine β-synthase, *mccA*; cystathionine γ-lyase, *mccB*. The following genes are absent from the genome of *C. perfringens*: *metI *(cystathionine β-synthase); *metC *(cystathionine β-lyase); *metE *(methionine synthase). AI-2, autoinducer 2; OAS, *O*-acetyl-serine; SAM, S-adenosyl-methionine; SAH, S-adenosyl-homocysteine; SRH, S-ribosyl-homocysteine. Ext means external.

Sulfur metabolism is well characterized in *Bacillus subtilis *[6]. In this bacterium, cysteine is synthesized either from homocysteine via the reverse transsulfuration pathway or from sulfide or thiosulfate via the thiolation pathway that directly incorporates these compounds into *O*-acetyl-L-serine (OAS). Sulfide is obtained from the transport and reduction of inorganic sulfate. CysE, the serine acetyltransferase produces OAS from acetyl-CoA and serine while the OAS-thiol-lyase, CysK, further condenses sulfide and OAS to form cysteine [7]. An efficient conversion of methionine into cysteine is also observed in *B. subtilis *through the SAM recycling pathway and then the reverse transsulfuration pathway (Fig. 1) that requires the sequential action of cystathionine β-synthase (MccA) and cystathionine γ-lyase (MccB) [8]. Cysteine is converted into methionine by the transsulfuration pathway followed by a methylation due to methionine synthases. In other firmicutes like *Bacillus cereus*, *Listeria monocytogenes *and several *Streptococci*, sulfide is directly converted into homocysteine by thiolation [9].

As a result of its crucial role in cellular physiology and the reactivity of the SH group of cysteine, sulfur metabolism is tightly controlled in response to environmental changes. Several molecular regulatory mechanisms have been identified in firmicutes. This includes regulation by premature termination of transcription at S-box and T-box systems responding to SAM pools and to the level of charge of tRNA, respectively [10,11]. LysR-type transcriptional regulators are also involved in the control of sulfur metabolism: CysL and YtlI in *B. subtilis *[12,13], CmbR in *Lactococcus lactis *and CysR and MetR/MtaR in *Streptococci *[14,15]. In *B. subtilis *and *Staphylococcus aureus*, the CymR repressor is the master regulator of cysteine metabolism [16,17]. CymR and CysK, the OAS-thiol-lyase, form a regulatory complex. CymR is the DNA binding protein while CysK increases the stability of CymR bound to DNA. In the signal transduction pathway controlling cysteine metabolism, CysK, via its substrate OAS, is the sensor of the cysteine pool in the cell for the regulatory complex [18].

As compared with other firmicutes, little is known about the sulfur metabolism and its regulation in the spore forming anaerobic clostridia. We have recently identified an original mechanism of control of the *ubiGmccBA *operon involved in methionine to cysteine conversion in *Clostridium acetobutylicum*. This regulatory mechanism involves two systems of premature termination of transcription, a cysteine specific T-box and an S-box, as well as the formation of antisense RNAs [19]. The *cis*-acting antisense RNAs transcribed from the downstream S-box-dependent promoter play a central role in the regulation of *ubiG *transcription in response to methionine availability.

*Clostridium perfringens *is the causative agent of various diseases including gas gangrene and food poisoning. This bacterium produces numerous extracellular toxins [20,21]. In *C. perfringens *strain 13, the VirS/VirR two component system is involved in the coordinated regulation of production of several toxins: the alpha-toxin (*plc*), the theta-toxin (*pfoA*) and the kappa-toxin (*colA*) [22,23]. The response regulator VirR directly regulates the expression of *pfoA *and of three non-coding RNAs, the VR-RNA, VirU and VirT, which in turns control the expression of *plc *and *colA *[24-26]. Another small non-coding RNA, VirX regulates *pfoA*, *plc *and *colA *expression independently from the VirS/VirR system [27]. Interestingly, the expression of the *ubiGmccBAluxS *operon of *C. perfringens *is repressed by the two-component system VirS/VirR via the VR-RNA [26,28,29]. This suggested the existence of links between the regulatory cascade of virulence and sulfur metabolism in *C. perfringens*. We therefore decided to study the sulfur metabolism and its regulation. We combined metabolic reconstruction, growth assays and expression profiling to obtain a global view of the sulfur metabolic network in *C. perfringens*. By comparative transcriptome analysis, we showed that a large set of genes was differentially expressed in *C. perfringens *strain 13 after growth in the presence of homocysteine or cystine, the dimer of cysteine being used as sole sulfur source. Among them, cysteine biosynthesis and transport, [Fe-S] clusters biogenesis, PfoA production and lactate dehydrogenase were regulated in response to cysteine availability. Finally, we showed the involvement of cysteine specific T-boxes in the derepression of genes involved in cysteine uptake and biosynthesis during cysteine depletion.

## Methods

### Bacterial strains and culture conditions

In this study, we used the *C. perfringens *strain 13 and several mutants of this strain: TS133 (*virR*::*tet*), TS140 (Δ*vrr*::*erm*) and TS186 (Δ*virX*::*erm*) [25,27]. *C. perfringens *strain 13 and its derivatives were grown under anaerobic conditions (10% H_2_, 10% CO_2_, 80% N_2_) in a sulfur-free minimal medium. We prepared a medium containing per liter: 1.14 g Na_2_HPO_4_, 0.28 g KH_2_PO_4_, 0.25 g alanine, 2.5 g arginine, 0.5 g glycine, 0.5 g histidine, 0.5 g isoleucine, 0.5 g leucine, 0.25 g phenylalanine, 0.375 g serine, 0.5 g threonine, 0.375 g valine, 1 g aspartate, 1 g glutamate, 0.25 g tyrosine, 0.0174 g adenine, 0.01 g uracil [30]. The pH was adjusted to 7 with HCl and the medium was autoclaved at 105°C for 20 min. Salts were then added at the following concentrations: 1 mM MgCl_2_, 50 *μ*M MnCl_2_, 35 *μ*M FeCl_3 _and 300 *μ*M ZnCl_2_. We also added 0.1 g/L glucose, 1 g/L tryptophane and 10 ml/L of a 100 × solution containing per liter 2 mg biotin, 2 mg folic acid, 10 mg pyridoxine, 5 mg thiamine, 5 mg riboflavin, 5 mg nicotinic acid, 5 mg calcium pantothenate, 5 mg paraminobenzoic acid, 5 mg lipoic acid and 0.1 mg vitamin B12. Various sulfur sources were then added to this sulfur-free medium at the following concentration: 0.5 mM cystine, 1 mM homocysteine, 1 mM glutathione, 1 mM thiosulfate, 1 mM sulfite, 1 mM sulfide, 1 mM or 5 mM methionine. When needed, antibiotics were added at the following concentration: erythromycin 25 μg ml^-1 ^and tetracycline 25 μg ml^-1^.

### Enzyme assays and estimation of metabolite content

Zymogram was performed to detect homocysteine γ-lyase activity. Strains 13, TS133, TS140 and TS186 were grown in minimal medium in the presence of 1 mM homocysteine or 0.5 mM cystine. Cells were harvested in exponential phase. After protein extraction, 100 *μ*g of crude extracts was applied to a non-denaturing protein gel (12% Tris-Glycine gel). After electrophoresis, the gel was washed twice for 10 minutes in 50 ml of water and twice for 10 minutes in 50 ml of Tris-HCl (50 mM, pH 7.4). The gel was then incubated at 37°C for 2 h with 50 mM Tris-HCl (pH 7.4), 10 mM MgCl_2_, 10 mM homocysteine, 0.5 mM Pb(Ac)_2_, 5 mM dithiothreitol and 0.4 mM pyridoxal phosphate (PLP). H_2_S formed during the enzymatic reaction precipitated as insoluble PbS. We therefore detected homocysteine γ-lyase activity by precipitated PbS. The signal was quantified with the "quantity one" software (Bio-Rad, USA). Visual observation of H_2_S production was performed using lead-acetate paper (Macherey-Nagel) that turned black following the incubation for up to 3 h at 37°C.

Intracellular concentrations of amino acids and other ninhydrin-reactive compounds were estimated using high-pressure liquid chromatography (HPLC). Briefly, cells were suspended in a sulfosalicylic acid buffer (3% final concentration) and disrupted using a FastPrep apparatus (Bio101). Supernatant samples were analyzed by cation-exchange chromatography, followed by ninhydrin postcolumn derivatization as previously described [8]. Intracellular metabolite concentrations were estimated assuming a cell volume of 4 μl per mg of proteins or a *C. perfringens *intracellular volume of 3 μm^3 ^[31]. Metabolite concentration was estimated with the ratio between total quantity of a metabolite and the total cellular volume. The mean value is calculated from three independent experiments. A statistical Wilcoxon test was realized giving a p-value < 0.05.

### RNA isolation, Northern blot analysis and quantitative RT-PCR

We extracted total RNA from strains 13, TS133 or TS186 grown in minimal medium with 0.5 mM cystine or 1 mM homocysteine as sole sulfur source. Cells were harvested at an OD_600 nm _of 0.6 (homocysteine) or 0.8 (cystine) by centrifugation for 2 min at 4°C. The cells were first broken by shaking in a Fastprep apparatus (Bio101) for 2 × 30 sec in the presence of one gram of 0.1-mm diameter glass beads (Sigma), then treated with Trizol reagent, chloroform/isoamylalcohol and precipitated with isopropanol. The pellet was resuspended in 100 μL of TE buffer (Tris 10 mM, EDTA 0.1 mM).

For Northern blot analysis, 10 μg of total RNA was separated in a 1.5% denaturing agarose gel containing 2% formaldehyde, and transferred to Hybond-N^+ ^membrane (Amersham) in 20 × SSC buffer (3 M NaCl, 0.3 M sodium citrate pH 7). Prehybridization was carried out for 2 h at 68°C in 10 ml of prehybridization buffer ULTRAHyb (Ambion). Hybridization was performed overnight at 68°C in the same buffer in the presence of a single strand RNA [α-^32^P]-labeled probe. The probes were synthesized from a PCR product containing a T7 phage promoter sequence on one of its extremities. One probe is located in the 5' untranslated region of the *cysP2 *gene (-326 to -181 relative to the *cysP2 *translational start point) and the second probe hybridizes with the coding region of *cysP2 *(+71 to +299 relative to the *cysP2 *translational start point). 1 μg of each PCR product was used as a matrix for *in vitro *transcription reaction with phage T7 RNA polymerase, 0.5 mM each ATP, GTP, CTP, and 50 μCi of [α-^32^P]UTP using Maxiscript kit (Ambion). The probe was then treated with TURBO DNAse I and purified on "Nucaway spin column" (Ambion). After hybridization, membranes were washed twice for 5 min in 50 ml 2× SSC 0.1%SDS buffer and twice for 15 min in 50 ml 0.1 × SSC 0.1% SDS buffer. Verification of equal loading was achieved by measurement of RNA concentration by absorbance at 260 nm and by direct comparison of rRNA band intensities after staining by ethidium bromide ("Quantity One" software, Bio-Rad, USA). The transcript size was estimated by comparison with RNA molecular weight standards (Ambion).

For quantitative RT-PCR (qRT-PCR) experiments, one μg of total RNA was heated at 65°C for 5 min. After a slow cooling, cDNAs were synthesized for 1 h at 42°C with Superscript II Reverse Transcriptase (Invitrogen), and 1 pmol of hexamer oligonucleotide primers (pDN6, Roche). The reverse transcriptase was inactivated by incubation at 70°C for 15 min. Real-time quantitative PCR was performed twice in a 20 μl reaction volume containing 100 ng or 1 *μ*g of cDNAs, 12.75 μl of the SYBR PCR master mix (Applied Biosystems), and 400 nM of gene-specific primers. Amplification and detection were performed as previously described [19]. In each sample, the quantity of cDNAs of a gene was normalized to the quantity of cDNAs of *gyrA*, which is a stably expressed gene in our transcriptome experiments. The relative change in gene expression was recorded as the ratio of normalized target concentrations (ΔΔct) [32].

### Microarray design for the *C. perfringens *genome, DNA-array hybridization and data analysis

The *C. perfringens *strain 13 genome was obtained from EMBL database. Probe design for the microarray was performed using the OligoArray 2.0 software [33]. 2 or 3 oligonucleotides were designed for each 2706 genes. We could not design oligonucleotides for 17 genes. Agilent produced the microarrays. Probes were replicated twice on the array to reach a final density of 13814 probes per array. 536 positive controls and 1394 negative controls were also included. The description of the microarray design was submitted to the GEO database (accession number GPL9765).

Total RNA was extracted from cells of 4 independent cultures for each growth condition. RNA was labeled with either Cy3 or Cy5 fluorescent dye (GE healthcare) using the SuperScript Indirect cDNA labeling kit (Invitrogen) according to the manufacturer's recommendations. A mixture of 10 μg of RNA and of pdN6 primers (Roche) was heated to 70°C for 5 min and quickly chilled on ice. We then sequentially added: 1× first-strand buffer, dithiothreitol (20 mM), dNTP mix, RNase OUT and 1600 units of Superscript III reverse transcriptase in a total volume of 24 μl. The reaction was incubated 3 h at 42°C to generate cDNAs. After alkaline hydrolysis and neutralization, cDNAs were purified on SNAP columns (Invitrogen) and precipitated with ethanol. The cDNAs were then mixed with Cy3 or Cy5 dyes (GE healthcare), incubated 1 h at room temperature in the dark, and purified on SNAP columns. 200 pmol of Cy3 and Cy5-labeled cDNAs was mixed and concentrated with microcon (Millipore). Hybridization was performed in micro-chambers for 17 h at 65°C according to the manufacturer's recommendations. 8 differential hybridizations were performed and each RNA preparation was hybridized twice with a dye swap. The array was then washed successively with Gene Expression Wash Buffer 1 and 2 (Agilent). We realized arrays scanning with a GenePix 4200L dual-channel (635 nm and 532 nm) laser scanner (GenePix). The complete experimental data set was deposited in the GEO database with accession numbers GSM480613 to GSM480620. All slides were analyzed using R and limma software (Linear Model for Microarray Data) from Bioconductor project http://www.bioconductor.org. For each slide, we corrected background with the 'normexp' method [34], resulting in strictly positive values and reducing variability in the log ratios for genes with low levels of hybridization signal. Then, we normalized each slide with the 'loess' method [35]. In order to identify genes differentially expressed, we used the bayesian adjusted t-statistics and we performed a multiple testing correction of Benjamini & Hochberg [36] based on the false discovery rate. A gene was considered as differentially expressed when the p-value is < 0.05.

### Stress response analysis

Disk diffusion assays were performed as follows: 20 ml calibrated agar plates were poured on a horizontal plane. *C. perfringens *strain 13 was grown in minimal medium containing 0.5 mM cystine or 1 mM homocysteine until it reached an OD_600 nm _of 0.5. The cells were then spread onto solid minimal medium containing the same sulfur source. After absorption, a sterile 6 mm disk was placed on the agar and 10 μl of 1 M H_2_O_2_, 1 M diamide or 0.2 M paraquat was added to the disk. The plates were incubated 48 h at 37°C and the diameters of growth inhibition were measured. These experiments were repeated 5-fold and a Wilcoxon test was realized giving a p-value < 0.05.

## Results and Discussion

### Reconstruction of sulfur metabolism in *C. perfringens*

**Figure 2:**
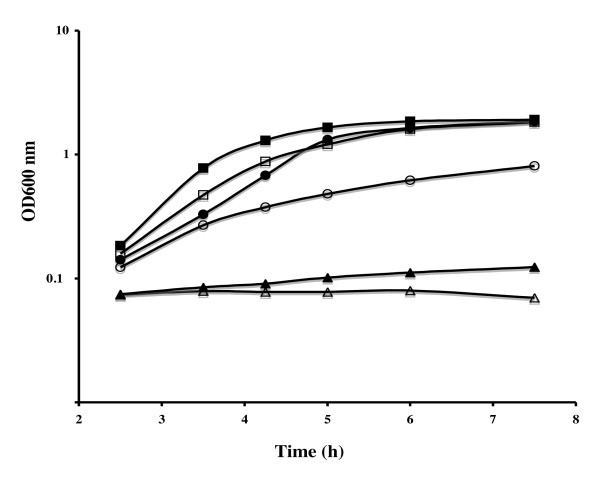
**Growth of *C. perfringens *strain 13 in the presence of various sulfur sources**. Growth curves of strain 13 grown in a sulfur-free minimal medium in the presence of 1 mM sulfite (■), 1 mM glutathione (□), 0.5 mM cystine (●), 1 mM homocysteine (○), 1 mM methionine (▲) or in the absence of any sulfur source (△). We observed a similar growth for homocysteine and cystathionine, thiosulfate and cystine or sulfide and sulfite.

We performed a systematic search in the *C. perfringens *genomes for genes known to be involved in assimilation pathways of sulfur-containing compounds. This tentative reconstruction is shown in Fig. 1. We also tested the ability of *C. perfringens *strain 13 to grow in a sulfur-free minimal medium in the presence of various sulfur sources in order to support the metabolic reconstruction performed and to obtain new insights about the physiology of this bacterium. We first tested the growth in the presence of the sulfur-containing amino-acids, methionine or cystine, the dimer of cysteine. This strain can grow in the presence of 0.5 mM cystine as sole sulfur source (Fig. 2) indicating a conversion of cysteine to methionine. Surprisingly, the genes required for methionine biosynthesis via transsulfuration or thiolation in other bacteria (*metA*, *metI*, *metC*, *patB*, *metY*, *metH*, *metE*, *metF*) [6,9] are absent in the genome of *C. perfringens *strain 13 [21]. This suggests the existence of an atypical methionine biosynthetic pathway in *C. perfringens*, which remains to be characterized.

Strain 13 cannot use methionine as sole sulfur source. This is intriguing since methionine can be converted into homocysteine by the SAM recycling pathway involving MtnN and LuxS and further to cysteine via the reverse transulfuration pathway probably encoded by the genes *cpe0176 *and *cpe0177 *(Fig. 1). We then tested the ability of strain 13 to grow in minimal medium containing 1 mM homocysteine or 1 mM cystathionine as sole sulfur source. We observed a growth with homocysteine and cystathionine indicating the existence of a pathway of homocysteine to cysteine conversion. Cpe0177 shares 51% and 70% identity with MccA, the cystathionine-β-synthase of *B. subtilis *and *C. acetobutylicum*, respectively while Cpe0176 is 56% and 70% identical to MccB, the cystathionine-γ-lyase/homocysteine-γ-lyase of the same microorganisms [8,19]. This strongly suggests that a reverse transsulfuration pathway is present in *C. perfringens *(Fig. 1) allowing the utilization of homocysteine, a compound that is present in human blood and tissues as an intermediary metabolite [37]. However, we cannot exclude the existence of another homocysteine to cysteine conversion pathway in *C. perfringens*.

The strain 13 was unable to grow on sulfate as sole sulfur source according to the lack of the first steps of the sulfate assimilation pathway. By contrast, strain 13 can grow in the presence of sulfite, sulfide or thiosulfate indicating that *C. perfringens *can synthesize cysteine from these compounds (Fig. 1 and 2). Sulfite is converted into sulfide by anaerobic sulfite reductases. Two operons, *asrABC1 *(*cpe1438-1440*) and *asrABC2 *(*cpe1536-1538*) encoding sulfite reductases are present in the genome. In the presence of sulfide and OAS produced by the serine acetyl-transferase (CysE), the OAS-thiol-lyase (CysK) further synthesizes cysteine. We tested the release of sulfide by the strain 13 after growth in the presence of various sulfur sources using lead acetate papers as a trapping agent. We detected high sulfide production after growth in the presence of sulfite due to sulfite reductase activities and to a lesser extent in the presence of thiosulfate. Sulfite and thiosulfate are taken-up by uncharacterized transporters since transporters sharing similarities neither with the CysPWUA system from *E. coli *[38] nor with the SA1850 permease from *S. aureus *[17] are present in the genome of *C. perfringens*. Thiosulfate is probably converted into cysteine using OAS-thiol-lyase activity as observed in *E. coli *[38]. Finally, *C. perfringens *was able to grow in the presence of glutathione. The PepT and PepM proteins could be involved in the degradation of this compound to form cysteine (Fig. 1). A pathway of glutathione synthesis from cysteine involving a bifunctional glutamate-cysteine ligase/glutathione synthetase is also present as previously proposed [39].

### Comparison of metabolite and gene expression profiles of *C. perfringens *grown with cystine or homocysteine

**Figure 3:**
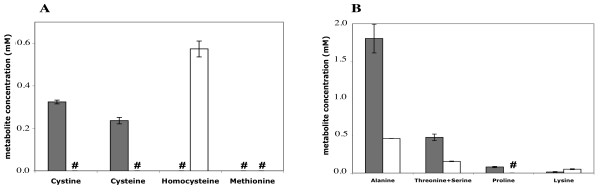
**Intracellular concentration of sulfur compounds (A) and amino acids (B) in strain 13 grown in the presence of cystine or homocysteine**. Grey or white boxes indicate the metabolite concentrations extracted from strain 13 grown in the presence of 0.5 mM cystine or 1 mM homocysteine, respectively. The mean value of three independent experiments is presented. # indicates that the metabolite is not detectable.

To obtain new insights into the regulation in response to sulfur availability, we compared the metabolome and the transcriptome of *C. perfringens *after growth in the presence of 0.5 mM cystine or 1 mM homocysteine. The doubling time was about two-fold higher for *C. perfringens *strain 13 grown in the presence of homocysteine than in the presence of cystine. Cystine allows efficient growth while homocysteine is a poor sulfur source for *C. perfringens*. This suggests that some metabolites are limiting during growth with homocysteine. So, we measured the intracellular concentration of several sulfur compounds and amino acids by HPLC in crude extracts of strain 13 grown in the presence of cystine or homocysteine (Fig. 3). The intracellular concentration of methionine remained undetectable in both growth conditions. This suggests that methionine biosynthesis is not very efficient and/or that methionine requirements are high. Homocysteine can be detected only during growth with this compound suggesting that homocysteine was mainly taken up from outside under these conditions. Cystine, cysteine but also proline pools were below the threshold of detection during growth with homocysteine while their intracellular concentrations were 325 μM, 236 μM and 80 μM, respectively during growth with cystine. This strongly suggests that growth in the presence of homocysteine mimics conditions typically associated with cysteine limitation. The concentration of alanine, lysine and serine and/or threonine differed to a lesser extent in these two conditions.

We further compared gene expression profiles of strain 13 grown in the presence of cystine or homocysteine. For this purpose, we designed a microarray containing oligonucleotides representative of 2706 genes of *C. perfringens*. For each condition, eight data sets generated with RNAs extracted from four independent cultures were used to perform statistical analysis (see Methods). A total number of 177 genes were differentially expressed in these two conditions. Most of them (122 out of 177) were up-regulated in the presence of homocysteine. Some of the controlled genes including those associated with sulfur metabolism, redox functions, carbon metabolism and virulence are presented in Table 1. We confirmed these transcriptome data by a qRT-PCR analysis using the same RNAs for a set of genes. About 20 genes of unknown function were also differentially expressed more than three-fold in response to cysteine availability in our transcriptomic data (Table 1). Except for *cpe2538*, all these genes were induced during conditions of cysteine limitation. Four genes (*cpe1078*, *cpe1386, cpe1387 *and *cpe1388*) encode cysteine-rich proteins. It was rather surprising to observe a drastic increase (6 to 11-fold) of synthesis of cysteine-rich proteins during cysteine limitation. Proteins required for sulfur assimilation, which are induced during conditions of sulfur starvation, are usually relatively depleted in sulfur-containing amino acids [40,41]. We will focus this paper on the genes involved in sulfur metabolism or functions with possible links with cysteine such as iron-sulfur cluster biogenesis and redox.

**Table 1:** Genes differentially expressed in strain 13 after growth in the presence of homocysteine or cystine.

Gene name (synonym)	Function/similarity	Transcriptome analysis		qRT-PCR	
		Homocysteine/cysteine	p-value	Homocysteine/cysteine	
**T-box_Cys _controlled genes**					
*cpe1321 *(*cysE*)	Serine acetyl-transferase	7.91	0.0001		
*cpe1322 *(*cysK*)	OAS-thiol-lyase	6.86	0.0002	120	
*cpe0967*	Na^+^-H^+^/Amino acid symporter	15.53	6.6E-06		
*cpe0947*	Na^+^-H^+^/Amino acid symporter	7.01	0.0002		
**S-box controlled genes**					
*cpe2177 *(*metK*)	SAM-synthase	2.7	0.015	14	
*cpe2317*	probable Na^+^-H^+ ^antiporter	1.4	0.01		
**Iron sulfur clusters**					
*cpe1786*	Rrf2-type regulator	3.41	0.0001	14	
*cpe1785 *(*iscS*)	Cysteine desulfurase	3.36	0.00027		
*cpe1784 *(*iscU*)	Iron sulfur cluster assembly	6.73	0.00008		
*cpe1783 *(*trmU*)	Methylaminomethyl-2-Thiouridylate-	3.5	< 1E-05		
	methyltransferase				
*cpe1469*	IscS-like protein	2.5	0.0009	8	
*cpe0664*	HesB-like protein	3.83	1.5E-05	11	
**Functions associated to redox**					
*cpe2511 *(*fer*)	Ferredoxin [3Fe-4S]	3.2	< 1E-05		
cpe777 (*rubR1*)	Rubredoxin	1.8	0.001		
*cpe0780 *(*rubR2*)	Rubredoxin	2.4	< 1E-05	4.3	
*cpe0778*	Probable flavohemoprotein	1.62	0.005		
*cpe1331 (rubY)*	Rubrerythrine	1.64	0.01		
*cpe*2447 (*fer*)	Ferredoxin 2[2Fe-2S]	0.52	0.01		
*cpe0782*	Alkyl hydrogen peroxide reductase	0.49	< 1E-05		
*cpe*2537	cytochrome c-type biogenesis protein	0.41	< 1E-05		
*cpe*2538	Unknown	0.25	3.5^E^-05		
**Carbon metabolism**					
*cpe2308*	Mannose-1-phosphate	3.5	2.3E-05		
	guanylyltransferase				
*cpe0103 (ldh)*	Lactate dehydrogenase	2.73	0.004	15	
**Transporters, membrane or exported proteins**					
*cpe2151*	Mercure-copper binding protein	5.1	< 1E-05		
*cpe1371*	Na^+^-dependent symporter	3.3	0.009	5	
*cpe0049*	Membrane protein	3.02	< 1E-05		
*cpe2456*	Membrane protein	2.84	1E-05		
*cpe0554*	Protein with signal sequence	2.74	0.0002		
*cpe0383*	Holin-like protein	2.6	0.004		
*cpe2595*	Na^+^/H^+ ^antiporter	0.34	< 1E-05		
**Virulence**					
*cpe0163*	Perfringolysin O	0.3	0.02	0.16	
*cpe1523 *(*nagL*)	Hyaluronidase	1.82	9.5E-05	2.3	
**Proteins of unknown function**					
*cpe1078*	Unknown (73 aa)	10.8	< 1E-05		
*cpe1079*	Unknown	7.77	3E-05		
*cpe1385*	Unknown	4.2	6E-05		
*cpe1386*	Unknown (85aa)	8.73	< 1E-05		
*cpe1387*	Unknown (71aa)	6.4	5E-06		
*cpe1388*	Unknown	5.94	5E-05		
*cpe0651*	Unknown	4.8	< 1E-05		
*cpe0015*	Unknown	4.7	2E-05		
*cpe0114*	Unknown (74 aa)	4.49	1.33E-05		
*cpe0113*	Unknown (75 aa)	3.98	6E-05		
*cpe0264*	Unknown (98 aa)	3.94	0.001		
*cpe2619*	Unknown (63 aa)	3.88	< 1E-05		
*cpe0067*	Unknown	3.6	1E-05		
*cpe0102*	Unknown (90 aa)	3.52	0.01		
*cpe1472*	Unknown	3.36	0.00735		
*cpe2037*	Unknown (89 aa)	3.16	0.0006		
*cpe0363*	Unknown (118 aa)	3.11	0.002		

The results presented are representative of 8 differential hybridizations using RNA preparations obtained from 4 independent cultures. aa means amino acids.

### Regulation of T-box controlled genes

**Figure 4:**
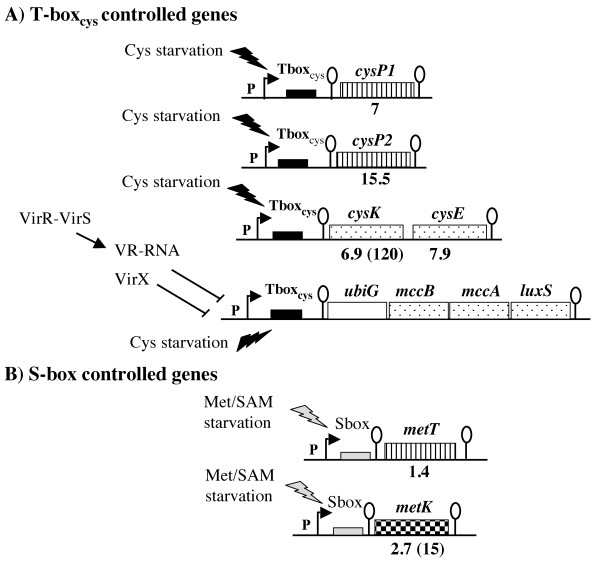
**Genes involved in sulfur metabolism controlled by premature termination of transcription via a T-box or an S-box system**. 5' untranslated region containing a T-box or an S-box motif are indicated by black or grey boxes, respectively. Loops indicate putative transcriptional terminators. Striped boxes indicate the genes encoding transporters. The genes involved in cysteine biosynthesis are indicated by dotted boxes while the SAM synthase gene, *metK*, is indicated by a checkerboard box. The expression ratios (homocysteine/cystine) obtained in transcriptome analysis are indicated under the genes while the expression ratios (homocysteine/cystine) obtained by qRT-PCR are indicated in parentheses. An alignment of the S-box motif of *metT *and *metK *has been previously published [9].

**Figure 5:**
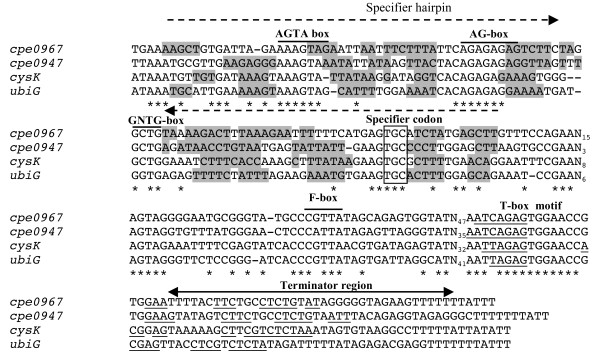
**Alignment of the 4 cysteine specific T-boxes present in the *C. perfringens *genome**. 4 genes with a T-box motif (AATTAGAGTGGAACC allowing one mismatch) were regulated in response to cysteine availability in transcriptome. We multialign a 200 bp region covering the T-box motif located upstream of these 4 genes. The conserved motifs characteristic of T-boxes (AGTA-box, F-box, AG-box, GNUG- box) are indicated. The cysteine specifier codon is boxed. Base-paired positions in the specifier hairpin (dotted arrow) are indicated by gray background. The bases involved in the formation of the antiterminator structure are underlined.

**Figure 6:**
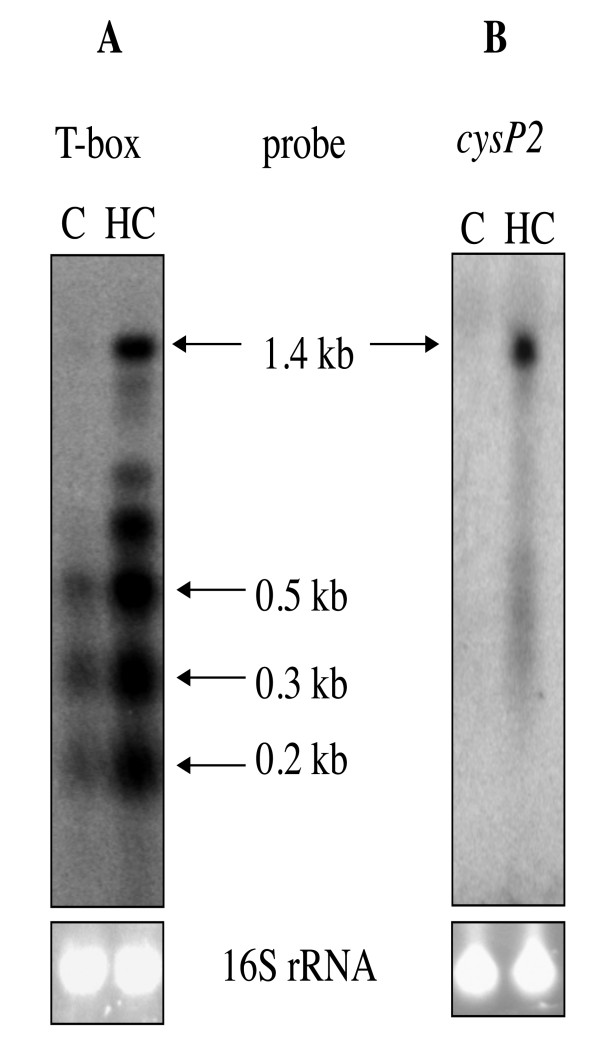
**Northern blot analysis of the T-box controlled *cysP2 *transcription**. Total RNA was extracted from strain 13 grown in minimal medium in the presence of cystine 1 mM (C) or homocysteine 1 mM (HC). Specific RNAs were detected using a probe hybridizing with the T-box region (A) or with the *cysP2 *(*cpe0967*) gene (B). The 16 S rRNA was used as a loading control.

Among genes derepressed after growth in the presence of homocysteine, we found genes encoding the serine acetyl-transferase, CysE, the OAS-thiol-lyase, CysK, and two transporters CysP1 (Cpe0947) and CysP2 (Cpe0967) (Fig. 1 and 4). T-box motifs are present upstream of *cysK*, *cysP1 *and *cysP2 *[42]. These T-box regulatory systems are mostly involved in the control of aminoacyl-tRNA synthetase genes but also of genes involved in amino-acid biosynthesis or uptake in firmicutes [11,42,43]. An alignment of the region surrounding the 15 bp T-box motif (AATTAGAGTGGAACC) located upstream of *cysK*, *cysP1 *and *cysP2 *is presented in Fig. 5. We clearly observed the presence of conserved AGTA-, AG-, GNUG- and F-boxes and a terminator downstream from the T-box motif with a possible alternative formation of an antiterminator structure. A specifier codon for cysteine (TGC) matching with the anticodon of the cysteinyl-tRNA is also present [11]. Interestingly, Vitreschak *et al *have shown that the TGC codon (100%) is preferred to the TGT codon at T-box regulatory sites [42]. The presence of a T-box specific for cysteine (T-box_Cys_) upstream of these genes is therefore in agreement with the 7 to 15.5-fold derepression under conditions of cysteine limitation in transcriptome. This strongly suggests that these genes are controlled by premature termination of transcription via a T-box element sensing cysteine availability. To confirm the control of *cysP2 *expression by premature termination of transcription, we performed Northern blot experiments using strand specific RNA probes located in the 5' untranslated region of the *cysP2 *gene (T-box region) or within its coding region. In the presence of cystine, we observed small transcripts of about 500, 300 and 200 bases with a probe hybridizing with the T-box region while no transcript was detected with the *cysP2 *probe (Fig. 6). The transcript of 300 bases has the size expected for a transcript initiated at a putative σA-dependent promoter located upstream of the specifier hairpin (data not shown) and terminated at the T-box terminator (Fig. 5 and 6) while the band at 200 bases might be due to RNA degradation or cleavage of the 300 base transcript as observed for other T-box elements [44]. In the presence of homocysteine, a larger transcript of about 1.4 kb, which corresponds to the transcription of the complete *cysP2 *gene appeared with both probes. As observed in transcriptome, the amount of *cysP2 *transcript drastically increases under conditions of cysteine depletion. Under these conditions, part of the transcriptional machinery passed through the terminator located upstream of *cysP2 *that contains a T-box_Cys _allowing *cysP2 *transcription (Fig. 4). CysP1 and CysP2 are Na^+^/H^+ ^symporters that could participate in the uptake of cysteine and/or cystine. These symporters share limited similarities with the cystine symporter TcyP of *B. subtilis *[45], and correspond to new classes of cyst(e)ine transporters [42]. CysP2-like proteins are present in the genome of other clostridia (*C. tetani*, *C. botulinum *and *C. novyi*). In addition to *cysP1 *and *cysP2*, the *cysK *and *cysE *genes that probably form an operon were co-regulated in response to cysteine availability via a T-box_Cys _located upstream of *cysK*. The expression of *cysK *was 7 and 120-fold higher during cysteine limitation in transcriptome and qRT-PCR experiments, respectively (Fig. 4). The expression of the *cysKE *operon increases during cysteine depletion in agreement with the involvement of CysK and CysE in cysteine biosynthesis.

**Figure 7:**
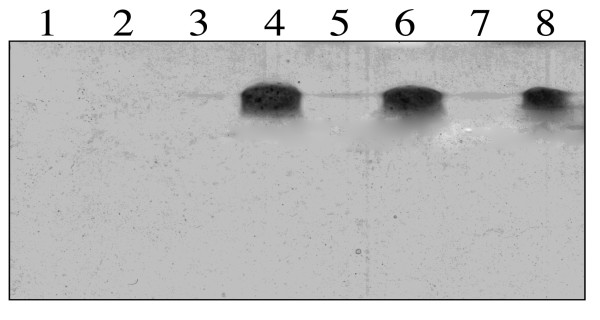
**Modulation of MccB synthesis in the presence of homocysteine or cystine in various mutants**. The homocysteine γ-lyase activity of MccB was detected on zymogram. A total of 100 *μ*g of crude extracts were charged on a native polyacrylamide gel (12%). The release of sulfide from homocysteine due to homocysteine γ-lyase activity was detected by the precipitation of insoluble PbS. The proteins were extracted from strain 13 (l and 2), TS140 (*vrr*::*erm*) (3 and 4), TS186 (*virX *::*erm*) (5 and 6) and TS133 (*virR*::*tet*) (7 and 8) after growth in the presence of 0.5 mM cystine (1, 3, 5 and 7) or 1 mM homocysteine (2, 4, 6 and 8).

Surprisingly, the *ubiGmccBAluxS *operon was not regulated in response to cysteine availability in transcriptome despite the presence upstream of *ubiG *of a T-box_Cys _element with all the conserved motifs of functional T-boxes (Fig. 5). MccB-type enzymes have both cystathionine γ-lyase and homocysteine γ-lyase activities [8]. To demonstrate a possible repression of this operon by cysteine, we tested the homocysteine γ-lyase activity of MccB by zymogram (Fig. 7) [19]. Using crude extracts of strain 13 grown with 0.5 mM cystine or 1 mM homocysteine as sole sulfur source, the homocysteine γ-lyase activity of MccB cannot be detected (Fig. 7, lane 1 and 2). However, it has been previously shown that the master regulator of virulence, VirR via a small regulatory RNA, the VR-RNA, negatively regulates *ubiG *expression [28,46]. Thus, we tested the homocysteine γ-lyase activity in the strain 13 inactivated for the *virR *gene (TS133), the *vrr *gene encoding the VR-RNA (TS140) or the *virX *gene (TS186) encoding another regulatory RNA controlling toxin production [25,27]. We detected by zymogram the homocysteine γ-lyase activity of MccB in crude extracts of these 3 mutants (Fig. 7, lane 3-8). This activity was about 100-fold higher in crude extracts of strains grown in the presence of homocysteine than in the presence of cystine. We then realized a qRT-PCR analysis using oligonucleotides hybridizing with *ubiG*. With RNAs extracted from TS133 (*virR*::*tet*), TS140 (*vrr*::*tet*), or TS186 (*virX*::*erm*), *ubiG *expression is respectively 45-, 67- and 250-fold greater in the presence of homocysteine than in the presence of cystine. This confirmed the results obtained with MccB activity and indicated that *ubiG *transcription drastically increased during cysteine depletion in the tested mutants. The cysteine specific T-box system is very likely involved in the induction of expression of the *ubiG *operon involved in sulfur metabolism and AI-2 production during cysteine limitation. Actually, a T-box_Cys _is also present upstream of the *ubiGmccBluxSmccA *operon of *C. botulinum *and the *ubiGmccBA *operon of *C. acetobutylicum *[9]. However, the regulation of *ubiG *expression in *C. perfringens *and *C. acetobutylicum *seems to differ. In *C. acetobutylicum*, the T-box_Cys _is not fully functional and the control of the *ubiG *operon involves mainly antisense RNAs whose expression is repressed in the presence of methionine via an S-box riboswitch [19].

MccB belongs to a family of PLP-dependent enzymes with *O*-acyl-homoserine γ-synthase, cystathionine β-lyase, cystathionine γ-lyase, methionine γ-lyase or *O*-acyl-homoserine thiol-lyase activity [47]. Several elements strongly support that Cpe0176/MccB is involved in reverse transsulfuration: i) MccB is more similar to characterized cystathionine γ-lyases of *B. subtilis *and *C. acetobutylicum *than to the other members of this family; ii) MccB has an homocysteine γ-lyase activity associated with cystathionine-γ-lyase activity [8]; iii) *mccB *is in operon with *mccA *encoding a cystathionine-β-synthase-type enzyme and *ubiG*, encoding a SAM-dependent methyl-transferase as observed in several firmicutes [8,9,19]; iv) *C. perfringens *can grow in the presence of homocysteine as sole sulfur source; v) the expression of the *ubiG *operon is induced by cysteine depletion via a cysteine specific T-box element as expected for a cysteine biosynthetic pathway. In addition to its control by a T-box regulatory element, the *ubiG *operon also belongs to the VirR and VirX regulons. Interestingly, we showed that another member of the VirR and VirX regulons, the *pfoA *gene encoding perfringolysin O [24,27], was regulated in response to cysteine availability. *pfoA *expression increased 3- (transcriptome) and 6-fold (qRT-PCR) in the presence of cystine compared with homocysteine (Table 1). However, it seems unlikely that the effect of cysteine is mediated by the VirR/VirS system since cysteine does not induce the expression of other VirR/VirS-activated genes [48].

### Regulation of other genes involved in sulfur metabolism by cysteine availability

An S-box motif is located upstream of two genes that were derepressed during cysteine depletion in the transcriptome study: the *metK *gene encoding the SAM-synthase and the *cpe2317 *gene (*metT*) encoding a potential methionine transporter [9] (Fig. 1). Cpe2317/MetT is an antiporter of the NhaC superfamily that is present in *B. cereus*, *S. aureus*, *C. botulinum *and *C. tetani *with S-boxes preceding the corresponding genes [9]. Quantitative RT-PCR experiments confirmed that the quantity of the *metK *transcript was 14-fold higher in the presence of homocysteine than in the presence of cystine. This suggested that the concentration of SAM is limited during growth with homocysteine. We were unable to detect methionine (Fig. 3) suggesting a low concentration for this amino acid. We also failed to reproducibly determine the SAM concentration probably due to the weak stability of this compound.

In this study, we identified additional genes that could participate in sulfur metabolism. We observed an increased transcription of *cpe1371 *in the presence of homocysteine (3.3-fold in transcriptome and 5-fold in qRT-PCR experiments). This gene encodes a Na^+^-dependent symporter of amino acids that could participate in the uptake of sulfur-containing compounds (cystine, cysteine, homocysteine, S-methyl-cysteine, glutathione). By contrast, the *asrABC1 *and *asrABC2 *operons as well as the *pepT *and *pepM *genes (Fig. 1) were not differentially expressed after growth in the presence of homocysteine or cystine. The synthesis of sulfite reductases may be induced in the presence of sulfite as shown for *Clostridium pasterianum *[49]. In the absence of sulfite in the growth medium, we do not observe any regulation for the *asr *operons by the sulfur sources tested. Among the genes differentially expressed during cysteine depletion, we were also unable to identify candidates for methionine biosynthesis. The enzymes involved could be either constitutively synthesized or the effector modulating the transcription of the corresponding genes is not sufficiently depleted under the growth conditions tested.

### Control of iron-sulfur cluster biogenesis and related functions

Expression of genes involved in [Fe-S] cluster biogenesis was regulated in response to cysteine availability (Table 1). Actually, four genes adjacent on the chromosome, *cpe1783 *to *cpe1786*, were up-regulated 3 to 6-fold during cysteine limitation. Cpe1786 is a repressor of the Rrf2 family sharing 50% identity with CymR, the global regulator of cysteine metabolism of *B. subtilis *[16] and 37% with IscR, the regulator of [Fe-S] cluster biogenesis in *E. coli *[50]. Cpe1785 and Cpe1784 encode a cysteine desulfurase and a scaffold protein for [Fe-S] cluster assembly, respectively [1] while TrmU (Cpe1783) is an enzyme involved in thio-uridylation of tRNAs. In the absence of nitrogen fixation in *C. perfringens*, we proposed to rename *cpe1785*, *iscS *instead of *nifS *and *cpe1784*, *iscU *instead of *nifU*. The expression of *cpe1469 *encoding a putative cysteine desulfurase sharing 25% identity with IscS also increased during cysteine depletion. Finally, the expression of *cpe0664 *encoding a 114 amino-acid protein, which corresponds to an A-type carrier required for [Fe-S] cluster assembly [51], was induced during cysteine limitation (Table 1). Thus, in the absence of the *suf *genes in *C. perfringens*, *iscSU *and *cpe0664 *probably constitute the unique system of [Fe-S] cluster biogenesis in this bacterium [1]. In *E. coli *and several other bacteria, genes involved in this process are regulated in response to [Fe-S] availability via the [Fe-S] protein IscR, and are induced during iron starvation and oxidative stress [1,52]. By contrast, only few data are available concerning the control of [Fe-S] cluster synthesis by cysteine availability. The coordinated derepression of genes involved in [Fe-S] production (*cpe1785, cpe1784, cpe1469, cpe0664*) during cysteine depletion may allow *C. perfringens *maintaining its pools of [Fe-S] clusters, which play a crucial role in the physiology of these bacteria lacking the heme synthesis machinery [53].

Expression of *ldh *encoding the lactate dehydrogenase (LDH) increased 2.7-fold after growth in the presence of homocysteine as compared with cystine. Several [Fe-S] clusters containing enzymes (pyruvate-ferredoxin-oxidoreductase, ferredoxin, hydrogenase) participate in the production of acetyl-CoA from pyruvate in clostridia while lactate production by LDH does not require [Fe-S] clusters [53,54]. The conversion of pyruvate to acetyl-CoA is therefore dependent on the iron and cysteine supply. *C. perfringens *might increase LDH synthesis during cysteine limitation to decrease the excess of reducing equivalents produced by glycolysis combined with low [Fe-S] cluster requirements. Interestingly, the lactate production is increased during iron starvation in *C. acetobutylicum *[55].

### Regulation of genes involved in redox systems

Genes involved in electron transfer and maintenance of the cell redox status were also differentially expressed in response to cysteine availability. The expression of *cpe2511 *encoding a [3Fe-4S] ferredoxin was up-regulated in the presence of homocysteine while that of *cpe2447 *encoding a 2[2Fe-2S] ferredoxin playing a role in shuttling electrons between a number of redox enzymes [53] increased in the presence of cystine. The *rubR1 *and *rubR2 *genes encode rubredoxins. These proteins contain an iron associated to 4 cysteinyl residues and play a role in electron transfers for the nitrate reductase or the NADH/rubredoxin oxidoreductase involved in oxygen and reactive oxygen species detoxification [56,57]. The *rubR *genes were about 2-fold more expressed in the presence of homocysteine than in the presence of cystine. We confirmed by qRT-PCR that *rubR2 *was 4-fold up-regulated during cysteine limitation. The rubredoxins participate in the oxidative stress response in *C. perfringens *and *C. acetobutylicum *[56,58] via their role in electron transfer for the NADH/rubredoxin oxidoreductase involved in the detoxification of oxygen and reactive oxygen species [59,60]. We then tested the sensitivity of strain 13 to stresses after growth in the presence of homocysteine or cystine. The growth inhibition area in the presence of H_2_O_2 _and diamide increased 11 and 13% (p-value <0.05), respectively in the presence of homocysteine as compared with cystine while no difference was observed with paraquat. So, the strain 13 appeared more sensitive to H_2_O_2 _and diamide during cysteine depletion despite the induction of *rubR1 *and *rubR2 *transcription. This induction is probably not sufficient to increase the resistance to H_2_O_2 _in the absence of induction of other scavenging components [NADH/rubredoxin oxidoreductase, FprA, Rubperoxin (formerly reverse rubrerythrin)]. The increased sensitivity of strain 13 to H_2_O_2 _and disulfide stress may be rather due to cysteine depletion during growth with homocysteine. Cysteine is a precursor of glutathione that is detected in *C perfringens *[61]. Glutathione plays a key role in thiol homeostasis and in protein protection after an oxidative stress [62]. However, genes involved in glutathione biosynthesis (Fig. 1) or encoding glutaredoxins are found in the genome of *C. perfringens *but proteins sharing similarities with glutaredoxin-reductases are lacking. The possible involvement of glutathione or other cysteine derivatives as a low-molecular-weight antioxidant in *C. perfringens *remains to be determined.

## Conclusion

Most of genes involved in sulfur metabolism in *C. perfringens *are controlled in response to sulfur availability by premature termination of transcription. An S-box motif is located upstream of the *metK *gene encoding a SAM synthase and the *metT *gene encoding a probable methionine transporter. Two pathways leading to cysteine production from methionine (LuxS, MccA, MccB) or sulfide (CysKE) and two cyst(e)ine transporters are controlled by a T-box_cys _regulatory element. By different approaches, we have demonstrated that the 4 cysteine specific T-boxes of *C. perfringens *respond to cysteine availability and that the T-box upstream of *cysP2 *promotes premature termination of transcription in the presence of cysteine. Interestingly, T-boxes are present upstream of the *ubiG *and *cysKE *operons and the *cysP2 *gene of *C. botulinum *[42] as well as the *cysKE *and *ubiG *operons of *C. kluyveri *suggesting conserved mechanisms for the control of cysteine metabolism in these clostridia. By contrast, no T-box is present upstream of *cysK *of *C. acetobutylicum, C. tetani *and *C. novyi *or *cysP2 *of *C. tetani *and *C. novyi *suggesting that other mechanisms of control of cysteine metabolism may exist in clostridia. In other firmicutes, cysteine specific T-boxes are mainly found upstream of *cysS *encoding the cysteinyl-tRNA synthetase or *cysES *while cysteine metabolism is controlled by CymR-type regulators in Bacillales and by CysR in Streptococci [16].

In *C. perfringens*, the expression of the *ubiG *operon involved in methionine to cysteine conversion and in AI-2 production is submitted to a complex regulatory network with a triple control: i) a drastic induction during cysteine starvation via the cysteine specific T-box system present upstream of *ubiG *that senses the level of charge of tRNA_cys _[11]; ii) a control by the VirS/VirR two-component system via the VR-RNA by a still uncharacterized mechanism and iii) a regulation by VirX, a regulatory RNA, which controls toxin production independently from VirR. The control of *ubiG *expression by global virulence regulators like VirR and VirX suggests a role of this operon during infection. Its control by VirR and VirX might allow i) maintaining the pool of methionine, an amino-acid that cannot be synthesized by human cells and/or ii) limiting the pool of cysteine, an amino-acid that promotes oxidative DNA damages by driving the Fenton reaction due to the efficient reduction of Fe^3+ ^by cysteine [63]. This may contribute to increased resistance to reactive oxygen species during infection.

Finally, several genes are up-regulated during cysteine depletion via mechanisms different from the T-box and S-box systems in *C. perfringens*. It is worth noting that among the genes induced during cysteine limitation, there is *cpe1786 *encoding a repressor of the Rrf2 family. Cpe1786 is a good candidate to participate in cysteine-dependent regulation of iron-sulfur clusters biogenesis but maybe also of some steps of fermentation pathways. This deserves further investigations.

## Authors' contributions

BD, KO, TS and IMV conceived and designed the experiments. GA, EH and MM performed the experiments. MM, BD, KO, TS and IMV analyzed the data. BD, TS and IMV wrote the paper. All authors read and approved the final manuscript.
